# Identification of quantitative trait loci for yield and yield related traits in groundnut (*Arachis hypogaea* L.) under different water regimes in Niger and Senegal

**DOI:** 10.1007/s10681-015-1472-6

**Published:** 2015-05-30

**Authors:** Issa Faye, Manish K. Pandey, Falalou Hamidou, Abhishek Rathore, Ousmane Ndoye, Vincent Vadez, Rajeev K. Varshney

**Affiliations:** Institut Sénégalais de Recherches Agricoles (ISRA)-Centre National de Recherches Agronomiques (CNRA), BP 53, Bambey, Senegal; International Crops Research Institute for the Semi-Arid Tropics (ICRISAT), Hyderabad, 502 324 India; International Crops Research Institute for the Semi-Arid Tropics (ICRISAT), Sahelian Center, 12404 Niamey, Niger; Department of Biology, Faculty of Sciences, University Abdou Moumouni, BP 10662, Niamey, Niger

**Keywords:** Drought tolerance, Epistatic QTLs, Main-effect QTLs, Pod yield, Quantitative trait locus

## Abstract

Yield under drought stress is a highly complex trait with large influence to even a minor fluctuation in the environmental conditions. Genomics-assisted breeding holds great promise for improving such complex traits more efficiently in less time, but requires markers associated with the trait of interest. In this context, a recombinant inbred line mapping population (TAG 24 × ICGV 86031) was used to identify markers associated with quantitative trait loci (QTLs) for yield and yield related traits at two important locations of West Africa under well watered and water stress conditions. Among the traits analyzed under WS condition, the harvest index (HI) and the haulm yield (HYLD) were positively correlated with the pod yield (PYLD) and showed intermediate broad sense heritability. QTL analysis using phenotyping and genotyping data resulted in identification of 52 QTLs. These QTLs had low phenotypic variance (<12 %) for all the nine traits namely plant height, primary branching, SPAD chlorophyll meter reading, percentage of sound mature kernels, 100 kernel weight, shelling percentage, HI, HYLD and PYLD. Interestingly, few QTLs identified in this study were also overlapped with previously reported QTLs detected for drought tolerance related traits identified earlier in Indian environmental conditions using the same mapping population. Accumulating these many small-effect QTLs into a single genetic background is nearly impossible through marker-assisted backcrossing and even marker-assisted recurrent selection. Under such circumstances, the deployment of genomic selection is the most appropriate approach for improving such complex traits with more precision and in less time.

**Electronic supplementary material:**

The online version of this article (doi:10.1007/s10681-015-1472-6) contains supplementary material, which is available to authorized users.

## Introduction

Groundnut or peanut (*Arachis hypogaea* L.), an important oilseed and confectionary crop, is cultivated in >100 countries of the world. It covers 24.6 million ha global area with the total harvest of 41.3 million tons and productivity of 1676 kg ha^−1^ (FAOSTAT 2014). The Asia (11.6 mha, 47.15 %) and the Africa (11.7 mha, 47.56 %) together (23.3 mha, 94.71 %) cover maximum global area. Despite development of several new improved cultivars together with available management practices, the Asia and the Africa could achieve mere productivity of 2217 and 929 kg ha^−1^, respectively as compared to the Americas (3632 kg ha^−1^) (FAOSTAT 2014). Engagement of mostly smallholder farmers in groundnut cultivation under rainfed condition with limited resources and input is the main reason behind poor performance. In addition, majority of the cultivated area falls under semi-arid tropic (SAT) regions which faces frequent erratic rainfall mostly during flowering and pod filling stages (Mahmoud et al. 1992; Fletcher et al. 1992). Groundnut, as a legume species, is no less important in the nutrition for grazing animals as it is for human. Thus, apart from pod yield, genotypes with biomass production are desirable in SAT zone, which can provide a source of additional revenue for farmers and important source of feed for animals. Niger and Senegal are the major contributors in West Africa and drought stress has been the main challenge in getting pod yield up to full genetic potential of popular and widely cultivated cultivars. Unpredictable environment due to climate change has further made the task of breeders very difficult to improve these complex traits.

Drought tolerance and pod yield are the two highly complex traits and genotype (G) × environment (E) plays critical role in deciding the ultimate pod yield achieved under drought stress. The complexity of drought is further aggravated in the SAT due to occurrence of high temperature, high levels of solar radiation and poor soil health. The above mentioned variables/factors have always posed difficulty in identifying the specific physiological traits needed to be improved for enhancing crop performance under drought stress condition. The breeding community has always relied on selections based on phenotyping i.e., pod yield achieved under drought stress in a specific environment. These genotypes not only failed to perform in other environments but also sometimes even comparable performance could not be achieved in farmers’ field in the same environment.

Understanding trait genetics and identification of genetic factors with stable performance across environments based on precise phenotyping is essential to harness the benefits of modern genomics technology and the process is referred as genomics-assisted breeding (GAB) (Varshney et al. 2005, 2006). Last decade witnessed sharp progress in development of large genomic resources in groundnut along with their successful utilization in molecular breeding for trait improvement. Molecular markers have already been deployed at large scale in several genetics and breeding applications in groundnut (Pandey et al. 2012; Varshney et al. 2013a, 2014). In order to integrate genomics in breeding, identification of genomic regions/genetic markers for component traits of pod yield and drought tolerance is essential. Identification of main-effect (M-QTLs) as well as epistatic QTLs (E-QTLs) is the key for GAB to improve above mentioned traits. Of the several successful examples of integration of genomics into breeding for improving drought tolerance, the most notable efforts include introgression of desirable QTL alleles for drought tolerance in rice (*Oryza sativa*) (Shen et al. 2001; Courtois 2007), QTLs for higher yield under drought stress in maize (*Zea mays* L.) (Ribaut et al. 2008) and “*QTL*-*hotspot*” harbouring QTLs for several drought tolerance traits in chickpea (*Cicer arietinum*) (Varshney et al. 2013b).

In the case of groundnut (*A. hypogaea* L.), despite presence of large morphological variation in germplasm accessions, low genetic variation was detected at the molecular level by DNA markers (Hopkins et al. 1999; Stalker and Mozingo 2001; He et al. 2003; Ferguson et al. 2004). During the last 5 years, several genetic maps were constructed and QTLs were identified for yield components and traits potentially related to drought adaptation (Varshney et al. 2009; Ravi et al. 2011; Fonceka et al. 2012; Gautami et al. 2012) including genome-wide association studies (Pandey et al. 2014). Even though many QTLs were identified in above mentioned studies but these QTLs had low phenotypic variance. The major bottleneck remains in introgression of large number of such QTLs. In addition, complex traits are highly influenced by environments and QTLs identified at one specific location may not be valid for another location with varied environmental conditions. Thus, the QTLs identified under Indian environmental conditions (Varshney et al. 2009; Ravi et al. 2011; Gautami et al. 2012; Pandey et al. 2014) may not hold true for other SAT regions such as Eastern and Southern Africa (ESA) and West and Central Africa (WCA). Therefore, identification of QTLs for drought component traits specific to West Africa such as Niger and Senegal, where drought stress is a challenge, will have greater implications on groundnut improvement. Therefore, this study was conducted to phenotype an existing recombinant inbred line (RIL) population (TAG 24 × ICGV 86031) for yield related traits and examine their relationship with yield under well watered (WW) and water stress (WS) conditions in Niger and Senegal, and then compare to QTLs previously identified in an Indian context. We examined reproductive characteristics (shelling percentage, maturity percentage and one hundred kernel weight) and biomass features (plant height and number of primary branches) related to haulm yield under normal and stress conditions. The genotyping and phenotyping data were used for conducting QTL analysis to identify location specific and common QTLs across locations. The identified environment specific QTLs and linked markers will improve breeding efficiency and reduce time duration for developing cultivars with enhanced stress resilience for West Africa.

## Materials and methods

### Plant material

A RIL mapping population derived from a cross TAG 24 × ICGV 86031 with 318 individuals had been phenotyped earlier at ICRISAT, Patancheru (India). The phenotyping data generated for several drought tolerance related traits at ICRISAT, Patancheru (India) revealed large variation for transpiration efficiency and its related traits (Krishnamurthy et al. 2007). Most interestingly, this population also holds uniqueness of being the basis for development of first SSR-based genetic map for cultivated groundnut and improved version of this map now possesses 191 mapped loci (Varshney et al. 2009; Ravi et al. 2011). This population was also used for QTL analysis based on the phenotyping data generated at ICRISAT, Patancheru (India). In the present study, multi-seasonal phenotyping data was generated to identify QTLs at two important locations situated in two different WCA countries i.e., Niger and Senegal. Realizing the huge variations in environmental conditions of these two locations, the soil features and environmental statistics were also recorded in detail to draw reliable inference from this study.

### Growth conditions

The population was evaluated at the ICRISAT Sahelian Centre in Sadore (Niger) during 2009 and 2010, and at Centre National de Recherches Agronomiques (CNRA) at Bambey (Senegal) during 2009. The experiment was conducted under WW and WS conditions in the field. Local varieties (drought tolerant and drought sensible) were included as checks to assess the severity/extent of drought stress prevailed in a particular location/season in all the field experiments.

In Niger, the experiment was conducted during the rainy season (2009 and 2010) at ICRISAT Sahelian Centre, Sadore (13°6′N; 2°2′E; altitude 549 m) at a late planting date (August–December) to avoid the bulk of rains. These experiments have been referred to as Sad09 and Sad10 further in this manuscript. It is important to note that the soils at Sadore were arenosols (World reference base) with low pH, low water holding capacity, low inherent soil fertility and organic matter content. Therefore, fertilizer NPK (15-15-15) at a rate of 200 kg ha^−1^ and farmyard manure (2000 kg ha^−1^) was applied after completion of field plowing in order to grow a healthy crop. In both the experiments, a total of 284 RILs along with five check varieties were evaluated (a total of 289 entries) in a 17 × 17 alpha lattice design (LSD) with four replications. The experimental plot was consisted of two rows of 2 m length (row to row 50 cm and plant to plant 10 cm) achieving plant density of 20 plants m^−1^.

In Senegal, the experiment was conducted towards the end of the rainy season 2009 (October–January 2010) at the ISRA-CNRA center, Bambey (14°42′N; 16°28′W; altitude 20 m), further referred to as Bam09 in this manuscript. The soil of the experiment had a sandy texture (910–940 g kg^−1^) with <55 g kg^−1^ clay content, 2.7–3.4 g kg^−1^ organic matter, 1.7–2.2 cmol kg^−1^ carbon exchange capacity and 6.5 water pH. The field was previously cultivated with pearl millet and plowed before sowing. In this experiment, no fertilizer was added at any stage of the crop. At Bambey, the trial was carried out with 294 RILs and six varieties as checks (a total of 300 entries). The test was conducted under two conditions i.e., well watered (WW) and water stress (WS) conditions. All 300 entries were planted in an alpha lattice design comprising 10 blocks and 30 plots per block with three replications. Each plot consisted of two rows of 2.10 m length (row to row 50 cm and plant to plant 15 cm) achieving the plant density of 13 plants m^−1^.

### Irrigation management for imposing stress

In Senegal (Sadore), planting of the mapping population was done at the end of August in 2009 and 2010. In these years, in the first 4 weeks after planting, the experimental station received 240 and 224 mm rain in 2009 and 2010, respectively. The experiment station did not receive any rain after 4 weeks of plant stand. In Senegal (Bambey), the experiment station did not receive any rain during the entire experiment. In brief, as none of the three experiments received any rain during the crucial growth stages of the plants, we had ideal condition for conducting experiments under well watered (WW) and water stress (WS) conditions.

The drought stress was imposed after flowering (i.e. at 35 days after sowing) by irrigating water stress (WS) plots alternatively (only once every two times) while the well watered (WW) plots were irrigated at regular interval. The first irrigation up to 40 mm was provided for all the plots (WW and WS) at the time of flowering. The following irrigation was supplied about 7 days later to only WW plots based on the estimated evapotranspiration. The third irrigation was supplied to all the plots (both WW and WS). The time interval for third irrigation was based on a leaf wilting assessment of the WS plots i.e., irrigation being supplied when the wilting score of a majority of WS plots reached a value of 3.0. The score of 3.0 corresponded to a stage when majority of the plants of most plots showed wilting symptom in the early afternoon hours of the day (Hamidou et al. 2012). The fourth irrigation was supplied to only WW experiment plots while the fifth irrigation supplied again to both WW and WS experimental plots. This scheme was followed until maturity. Following this irrigation scheme, the irrigation of WS plots was half of that in the WW plots after the initiation of the water stress.

### Phenotypic measurements

Number of primary branches (PBr) and plant height (PH) were recorded at 60 days after sowing (DAS) at Bambey. At this stage, it was assumed that maximum plant development had reached and hence corresponds to highest transpiration rate. Plant height was estimated by measuring the length of the main stem from the plant collar to the plant apex and the same plants were used to count the number of primary branches. Phenotype of each genotype per replication was estimated as the value mean of the two sampled plants.

The SPAD chlorophyll meter reading (SCMR) measures absorbance by plant tissues of wavelengths in the visible spectrum and serves as a measure of the relative internal concentration of chlorophyll a and b. The SCMR was recorded using SPAD-502 (Minolta Corp., Ramsey, NJ, USA) in all the experiments with two plants per plot and on two fully developed leaves per plant in all the replications. Each reading was recorded on each of the four leaflets, care being taken to avoid the midrib, and averaged to correct for possible non-homogeneous distribution of chlorophyll throughout the leaf (Sheshshayee et al. 2006). The SCMR was evaluated at 15 days after stress initiation, at this time, scoring of wilting symptoms recorded was three in plots under water stress conditions.

Harvesting was carried out at about 100 DAS. Haulms can be defined as that part of the plant remaining after removing the pods. Pod weight as well as the haulm weight was estimated after air-drying the total biomass collected from each plot. Pod yield (PYLD) and haulm yield (HYLD) were expressed in grams per square meter (g^−2^). Pod weight as well as the haulm weight was used to estimate the yield performance of each genotype in all the three experiments. The harvest index (HI) indicates the efficiency of translocation of assimilates from the vegetative tissues to reproductive tissues. The HI was computed as the ratio between pod yields over the total biomass of a given genotype.

In the experiment conducted at Bambey, yield components such as shelling percentage (SP), 100 kernels weight (100 KW) and the percentage of sound mature kernels (SMK %) were also recorded. All these traits were recorded after complete drying of pods in an oven at 65 °C. The percentage of maturity was based on the ratio between the numbers of mature pods out of 20 pods randomly sampled from each plot. The maturity of a pod was assessed by the blackening of the internal inner parenchyma (Miller and Burns 1971). The shelling percentage was estimated as the percentage by weight of kernels from 200 g of pods sampled from each replication. The 100 kernels weight was obtained by handpicked selections.

### Genotyping of mapping population with SSR markers

A total of 3215 simple sequence repeat (SSR) markers available in the public domain and/or accessed through collaborators were earlier used to screen the parental genotypes of this population. Genotyping was done with polymorphic markers for this population which resulted in development of an improved genetic map with 191 markers loci. The complete details on parental polymorphism, genotyping of mapping population and construction of genetic map is provided in Varshney et al. (2009) and Ravi et al. (2011).

### Statistical analysis of phenotypic assessments

All the phenotypic variables were tested for normal distribution. The iMAS ver 2.0 statistical package was used for analysis of variance (ANOVA) using a ReML (restricted maximum likelihood) mixed model analysis with RILs and replications × block as random effects. Broad sense heritability (*H*^*2*^) was computed under the same program as follow:

$$ H^{2} = \sigma^{2}_{\text{g}} /\sigma^{2}_{\text{g}} + \sigma^{2}_{\text{e}} $$

where $\sigma^{2}_{\text{g}}$ = genetic variance and $\sigma^{2}_{\text{e}}$ = residual variance.

The significance of genetic variance $\sigma^{2}_{\text{g}}$ is assuming the ratio $Z = {\text{s}}^{2}_{\text{g}} /{\text{S}}.{\text{E}}.({\text{s}}^{2} {\text{g}})$ to follow normal distribution asymptotically. Relationships between traits among environments were estimated by the Pearson correlation coefficients.

### Quantitative trait locus (QTL) analysis

QTL analysis was performed using existing genetic map information for 191 marker loci. Composite interval mapping (CIM) was conducted to identify main-effect QTLs (M-QTLs) and epistatic interaction analysis (EIA) to identify epistatic interactions between different genomic regions (epistatic-QTLs, E-QTLs) using WinQTL Cartographer, v. 2.5 (Wang et al. 2007) and QTLNetwork 2.0 (Yang et al. 2005), respectively. The threshold of LOD ≥2.5 was chosen for claiming putative QTL detected by WinQTL Cartographer. The mapping strategy, one-dimensional scan followed by two-dimensional search on the whole genome, was used for QTL detection with single-locus effect and epistasis effect, respectively. In each of the mapping procedures, permutation test was exploited to control genomewide false positive rate. Relative contribution was calculated as the proportion of variance caused by a specific genetic source in the total phenotypic variance.

In addition, EIA was also carried out using genotype matrix mapping (GMM 2.1) (Isobe et al. 2007, http://www.kajusa.or.jp/GMM) for identification of interactions between different loci. Two and three loci interactions were identified using GMM. All the QTLs (M- and E-QTLs) detected were anchored on the genetic map. All the M-QTLs identified and mapped were added a suffix which refers to the water regime (WW and WS), seasons (“09” refers to 2009 and “10” refers to 2010) and location (letter “B” for Bambey and “S” for Sadore).

## Results

The study identified several genomic regions (M-QTLs and E-QTLs) controlling yield component and drought tolerance related traits for Niger (Sadore) and Senegal (Bambey). The detailed information on the results is presented below.

### Climatic conditions of the field experiments

**Fig. 1:**
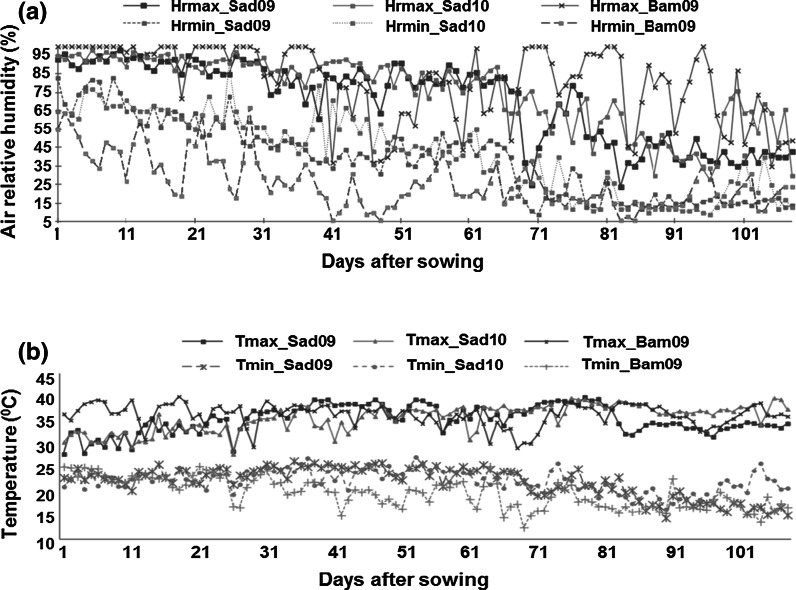
Environmental conditions at Bambey and Sadore during the period of experimentation. This figure shows the variation in (**a**) relative humidity in the air and (**b**) temperature during the crop period at Sadore and Bambey

Average minimum (T_min_) and maximum temperature (T_max_) in Sad09 and Sad10 were 22.1 ± 3.3 °C versus 22.3 ± 2.4 °C and 35.4 ± 2.8 °C versus 35.7 ± 2.7 °C, respectively. Similarly, average minimum (H_min_) and maximum relative humidity (H_max_) in Sad09 and Sad10 was 38.1 ± 20.8 % versus 40.8 ± 21.8 % and 69.6 ± 21.7 % versus 74.3 ± 19.5 %, respectively. At Bambey, the average minimum (T_min_) and maximum temperature (T_max_) were 19.7 ± 3.1 °C and 36.3 ± 2.3 °C, respectively while the average minimum (H_min_) and maximum relative humidity (H_max_) was 26.4 ± 13.8 % and 81.1 ± 19.9 %, respectively. Climatic conditions at Bambey were comparatively warmer than those at Sadore (Fig. 1). The number of days with maximum temperature (>35 °C) was higher at Bambey (83 days in 2009) than Sadore (59 days in 2009 and 73 days in 2010). At Sadore, weather condition during 2010 experiment was warmer than 2009. In addition to temperature, air relative humidity was drier at Bambey than Sadore (Fig. 1a and b).

### Phenotypic variation and broad-sense heritability

**Fig. 2:**
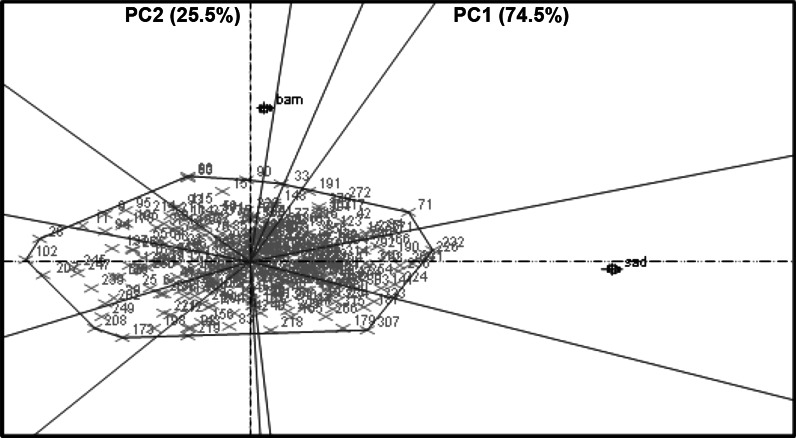
PC1 versus PC2 scatter plot of the pod yield under WS conditions at Bambey 2009 and Sadore 2009. This figure shows the comparison between two locations during the same year of experiment in pod yield

**Fig. 3:**
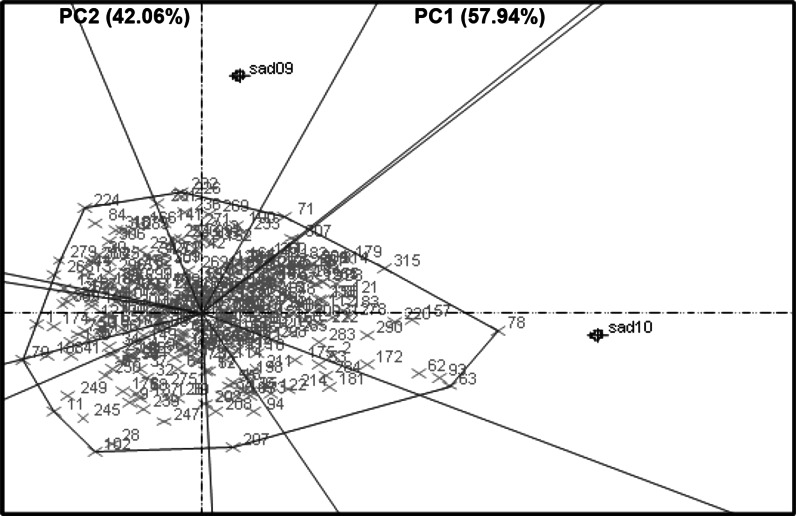
PC1 versus PC2 scatter plot of the pod yield under WS conditions at Sadore 2009 and Sadore 2010. This figure shows the comparison between two different year of experiment at a single location in pod yield

Large variation within the RIL population was observed for all the traits across the experiments (Table 1, Figs. 2, 3). For all the experiments under water stress conditions, a significant decrease in PYLD, HYLD and HI was observed while small or no decrease was observed for remaining traits. The ANOVA revealed significant genotypic components of variance (σ^2^g) and varied range of broad-sense heritability (*H*^*2*^) (0.11–0.73) for the RIL population depending on the trait, water regime and the experiment location (Table 1). At Bambey during 2009, the heritability of PYLD, HYLD, SCMR and HI was lower under WS than under WW while it was reverse at Sadore location during 2009 and 2010. The heritability of SP, 100 KW and SMK % was higher under WS condition than achieved under WW condition. Similarly at Sadore during 2009 and 2010, the heritability of the HYLD and SCMR was higher under WS condition than WW condition. The results for heritability of PYLD and HI were not consistent at Sadore during 2009 and 2010 in either of the water regimes.

**Table 1:** Grand mean, variation within the RIL and broad sense heritability (*H* ^2^) of each trait under well watered and water stress conditions in the different site

Site	Trait	Grand mean		Variation in RILs		Broad-sense heritability (*H* ^2^)	
		WW	WS	WW	WS	WW	WS
Bambey 2009	PYLD (g^−2^)	349.3	289.1	245.8–530.1	248.2–331.7	0.50	0.20
	HYLD (g^−2^)	425.5	341.4	227.8–765.8	238.4–448.9	0.72	0.37
	HI	0.46	0.46	0.33–0.55	0.39–0.51	0.65	0.23
	SCMR	45.5	44.3	41.36–49.97	40.51–47.65	0.33	0.20
	SP (%)	70.1	71.9	62.19–72.36	62.49–76.76	0.16	0.45
	SMK (%)	61.1	74.4	49.19–70.32	51.84–84.94	0.13	0.30
	100 KW (g)	68.7	69.3	62.45–121.00	53.46–84.33	0.11	0.40
	PH (cm)	14.72		7.20–22.15	–	0.73	–
	PBr	6.86		4.86–9.51	–	0.47	–
Sadore 2009	PYLD (g^−2^)	342.2	199.7	268.7–442.6	126.6–256.9	0.25	0.37
	HYLD (g^−2^)	412.9	394.7	234.4–682.5	231.5–554.4	0.62	0.48
	HI	0.57	0.45	0.41–0.70	0.18–0.66	0.57	0.59
	SCMR	38.3	38.9	31.7–42.4	31.7–38.9	0.33	0.25
Sadore 2010	PYLD (g^−2^)	303.1	190.8	203.8–456.7	112.8–320.1	0.22	0.30
	HYLD (g^−2^)	245.3	189.8	119.9–453.1	70.26–396.40	0.37	0.45
	HI	0.67	0.63	0.53–0.75	0.44–0.74	0.39	0.44
	SCMR	40.6	43.8	36.2–46.41	39.51–48.04	0.26	0.27

### Phenotypic correlations and factors contributing to pod yield

Under WW water regime, a total of 14 significant associations among nine traits were identified at Bambey during 2009 (Table 2). Of these significant associations, five associations were negative and nine significant associations were positive. The highest positive and significant association between different traits was 0.49*** (PYLD and HYLD, HI and SP) followed by 0.46*** (HYLD and PH) and 0.36*** (HI and SMK %). The highest negative and significant association was −0.71*** (HYLD and HI) followed by −0.43*** (HYLD and SMK %) and −0.42*** (HI and PH). At Sadore during both seasons (2009, 2010), the strongest positive association was between HYLD and PYLD (0.29***, 0.47***) while negative association between HYLD and HI (−0.78***, −0.47***) (Table 3).

**Table 2:** Pearson correlation coefficients between the different traits under well watered and water stress conditions at Bambey (CNRA 2009)

Water regime/Trait	Well watered (WW)										Water stress (WS)						
	PYLD	HYLD	HI	100 (KW)	SM (K %)	SCMR	SP	PH	PBr		PYLD	HYLD	HI	100 (KW)	SM (K %)	SCMR	SP
*Well watered (WW)*																	
PYLD	1																
HYLD	0.49***	1															
HI	ns	–0.71 ***	1														
100 KW	ns	0.11	ns	1													
SMK %	ns	–0.43***	0.36***	ns	1												
SCMR	ns	ns	ns	ns	ns	1											
SP	ns	ns	0.49***	ns	0.30**	ns	1										
PH	0.20*	0.46***	−0.42***	0.19*	ns	ns	−0.25**	1									
PBr	0.25**	0.27**	ns	ns	−0.24*	ns	ns	ns	1								
*Water stress (WS)*																	
PYLD	0.27**	ns	ns	ns	ns	ns	ns	ns	0.21*		1						
HYLD	0.24*	0.60***	−0.51***	ns	−0.31**	ns	−0.24*	0.35***	0.25**		0.24*	1					
HI	ns	−0.45***	0.49***	ns	0.22*	ns	0.27**	−0.38***	ns		0.41***	−0.73***	1				
100 KW	0.20*	ns	ns	0.38***	−0.25**	ns	ns	0.19*	ns		ns	0.22*	ns	1			
SMK %	ns	ns	ns	ns	ns	ns	ns	ns	ns		ns	−0.21*	ns	ns	1		
SCMR	ns	ns	ns	ns	ns	0.31**	ns	ns	ns		ns	ns	ns	ns	ns	1	
SP	ns	−0.45***	0.45***	ns	0.26**	ns	0.49***	−0.32**	ns		ns	−0.40***	0.43***	ns	ns	ns	1

*, **, ***, significant at probability P ≤ 0.05; P ≤ 0.01 and P ≤ 0.001, respectively

**Table 3:** Pearson correlation coefficients between the different traits under well watered and water stress conditions at Sadore (ICRISAT) during 2009 and 2010

Water regime	Trait	Well watered (WW)					Water stress (WS)			
		PYLD	HYLD	HI	SCMR		PYLD	HYLD	HI	SCMR
WW 2009	PYLD	1								
	HYLD	0.29**	1							
	HI	0.25**	−0.78***	1						
	SCMR	ns	ns	ns	1					
WS 2009	PYLD	0.47***	ns	0.33	ns					
							1			
	HYLD	ns	0.58***	−0.59***	ns		ns	1		
	HI	0.33***	−0.71***	0.86***	ns		0.42***	−0.64***	1	
	SCMR	ns	ns	ns	0.61***		ns	ns	−0.65***	1
WW 2010	PYLD	1								
	HYLD	0.69***	1							
	HI	ns	−0.47***	1						
	SCMR	ns	ns	ns	1					
WS 2010	PYLD	0.52***	0.40***	ns	ns		1			
	HYLD	0.45***	0.71***	−0.52***	ns		0.65***	1		
	HI	ns	−0.42***	0.61***	ns		ns	−0.45***	1	
	SCMR	ns	ns	ns	0.48***		ns	ns	ns	1

*, **, ***, significant at probability P ≤ 0.05; P ≤ 0.01 and P ≤ 0.001, respectively

Under WS water regime, only seven associations (four positive and three negative) could be detected for seven traits at Bambey during 2009 (Table 2). The strongest positive association was observed between HI and PYLD (0.41***), and HI and SP (0.43***) while strongest negative association was between HYLD and HI (−0.73***) and HYLD and SP (−0.40***). At Sadore during both seasons (2009, 2010), the strongest positive association was between HI and PYLD (0.42***, 0.65***) while negative association between HYLD and HI (−0.64***, −0.45***) (Table 3).

Upon comparing trait association between two water regimes, a total of 24 significant associations (16 positive and 8 negative) were identified at Bambey during 2009 (Table 2). The strongest positive association was found between HYLD (WW) and HYLD (WS) (0.60***) while negative association between HYLD (WS) and HI (WW) (−0.51***). Similarly at Sadore during both seasons (2009, 2010), the strongest positive associations were between HI (WW) and HI (WS) (0.86***, 0.61***) followed by HYLD (WW) and HYLD (WS) (0.58***, 0.71***) while negative associations between HI (WS) and HYLD (WW) (−0.71***, −0.42***) followed by HI (WW) and HYLD (WS) (−0.59***, −0.52***) (Table 3).

### Identification of location specific QTLs

#### Main-effect QTLs (M-QTLs)

**Fig. 4:**
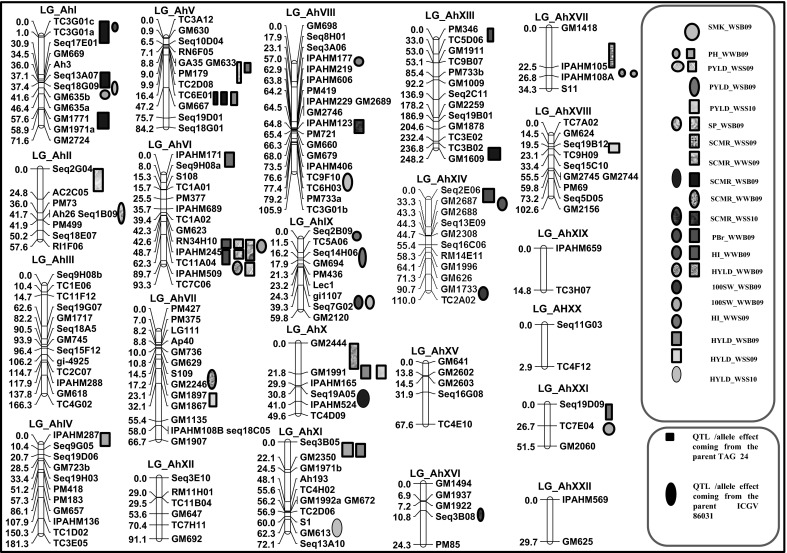
Genomic locations of main-effect QTLs (M-QTLs) identified in Sadore and Bambey for yield and yield related traits under well watered and water stress conditions. This figure shows the location of the mapped SSR loci on the genetic map of TAG 24 × ICGV 86031. The location of the M-QTLs identified by WinQTL Cartographer for different traits under well watered and water stress conditions at Bambey and Sadore were identified on the genetic map

QTL analysis resulted in identification of a total of 51 main-effect QTLs (M-QTLs) for nine different traits with phenotypic variance explained (PVE) ranging from 0.04 (PBr) to 11.56 %(PH) (Table 4; Fig. 4; ESM 1). Of the 51 M-QTLs detected, 27 M-QTLs were identified under WS condition and 24 under WW condition. Among locations, 38 M-QTLs were detected at Bambey, nine M-QTLs at Sadore during 2009 and four M-QTLs at Sadore during the experiment conducted in 2010.

**Table 4:** Main-effect QTLs (M-QTLs) identified under well watered and water stress conditions at Bambey and Sadore

Location and year	Trait	Well watered (WW)			Water stress (WS)	
		No. of QTLs	Range of PVE (R^2^ %)		No. of QTLs	Range of PVE (R^2^ %)
Bambey 2009	PYLD	–	–		1	7.51
	HYLD	3	3.74–6.96		3	5.33–10.00
	HI	3	3.50–8.28		–	–
	SCMR	1	4.96		7	2.96–8.11
	SP	–	–		2	5.74–6.97
	SMK %	4	3.50–7.41		2	3.30–3.85
	100 KW	1	8.78		1	11.56
	PH	5	4.04–8.16		NA	
	PBr	5	0.04–8.58		NA	
Sadore 2009	PYLD	–	–		4	5.16–11.38
	HYLD	–	–		2	7.70–8.20
	HI	1	3.77		–	–
	SCMR	1	3.98		1	9.59
Sadore 2010	PYLD	–	–		1	4.27
	HYLD	–	–		1	6.12
	HI	–	–		–	–
	SCMR	–	–		2	6.51–10.40

Of the five M-QTLs detected for PYLD under WS, four QTLs were detected at Sadore and one QTL at Bambey during 2009 (Table 4; Fig. 4; ESM 1). The PVE explained by these QTLs ranged from 5.16 to 11.3 %. For HYLD, a total of six QTLs were detected under WS condition i.e., three QTLs at Bambey during 2009, two at Sadore during 2009 and one at Sadore during 2010. Collectively, QTLs detected under WS accounted for 41.7 % of PVE for HYLD. Under WW condition, only three QTLs associated with HYLD were identified at Bambey during 2009 with low PVE. For HI under WW condition, three QTLs were identified at Bambey during 2009 and one QTL at Sadore during 2009 while no QTL was detected under WS condition in any location. Of the four QTLs detected for HI under WW, three QTLs were detected in Bambey alone. Of the 12 QTLs identified for SCMR under both the water regimes, 10 different QTLs were identified under WS condition and two QTLs under WW condition (one QTL each at Sadore during 2009 and 2010). Eight QTLs for SCMR were identified at Bambey with PVE ranging from 2.96 to 8.11 %. Two QTLs related to SCMR under WW were found i.e., one each at Bambey and Sadore 2009 with PVE range of 3.98–4.95 %.

In addition to three important traits explained above (PYLD, HYLD, HI and SCMR), five agronomical traits (SMK %, SP, 100 KW, PH and PBr) were also studied under both water regimes. A total of four QTLs were detected under WW and two QTLs under WS water regime for SMK % at Bambey during 2009. Two QTLs were detected for SP under WS condition while two QTLs were detected for 100 KW (one under each water regime). The QTL for 100 KW under WS exhibited the highest PVE (11.56 %) compared to remaining QTLs detected in this study. Further, five QTLs were detected for each of the PBr and PH in WW with PVE ranging from 0.004 to 0.856 % and 4.04–8.16 %, respectively.

#### Epistatic effect QTLs (E-QTLs)

With the QTLNetwork software, a single E-QTL was detected for HYLD at Sadore during 2010 between a locus on LG_AhIII (TC4G02) and LG_AhXVI (Seq 3B08) with 8.0 % PVE. Similarly, single E-QTL for SCMR was identified at Bambey 2009 between locus on the LG_AhI (Seq9H08b) and LG_AhXIII (PM346) with 4.98 % PVE. For the same trait at Sadore 2009, single E-QTL with flanking markers GM1009 and GM1878 mapped on the LG_AhXIII showed low PVE (3.84 %). Another E-QTL detected in the same environment was observed between two loci (Seq19B01 and TC3E02) of LG_AhXIII with 5.12 % PVE. Similarly, a single E-QTL for the SP was detected between the loci GM629 mapped on LG_AhVII and the loci GM2444 mapped on LG_AhX with 6.41 % PVE. Only single two-loci interaction between locus TC3G01a and IPAHM524 was identified for the PBr under WW at Bambey during 2009. A total of three E-QTLs were detected for PH located on LG_AhX and LG_AhXIV with low PVE (0.03–4.48 %).

**Fig. 5:**
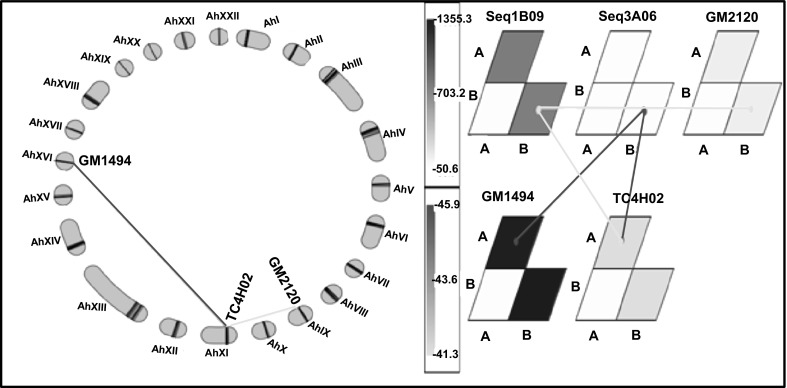
Epistasic QTLs (E-QTLs) for the pod yield detected at Sadore in 2009 using genotyping mapping matrix program. This figure shows the three-loci interaction detected for pod yield at Sadore in 2009. **a** Linkage groups are arranged tandemly as a *circle* and *triangles* in the *circle* represent interaction of three loci combination. **b** Graphical presentation of interacting loci and allele type by genotype matrices (GMs) and a genotype matrix network (GMN). Significant locus/allele combinations of three interacting loci are shown by GMs and GMN. Matrices and connecting lines indicate GMs and GMNs, respectively

With the GMM software, a two-loci interaction for SCMR (between a locus on LG_AhI and a locus on LG_AhXIII) detected by the QTL Network program was also detected with the GMM program. With this later program, two three-loci interactions were detected for the HYLD at Sadore during 2010. Among these interactions, single three-loci interaction involved one locus each mapped on LG_AhIII (gi-4925), LG_AhV (GM633) and LG_AhVIII (IPAHM229). The second three-loci interaction involved the loci (gi-4925, IPAHM229 and RN6F05) mapped on LG_AhV. For SCMR at Bambey during 2009, a single E-QTL identified by QTL Network was confirmed with the GMM. In addition to that E-QTL, a three-loci interaction was observed between loci mapped on LG_AhV, LG_AhXI and LG_AhXX. For the same trait but measured at Sadore during 2009, the GMM identified three different three-loci interactions and all these E-QTLs were different to those identified for SCMR at Bambey during 2009. On the contrary to the QTLNetwork program which identified no QTL, GMM program revealed two three-loci epistasic interactions for the PYLD at Sadore during 2009 (Fig. 5). In addition, GMM also revealed single two-loci interaction and two three-loci interactions for PBr while a single three-loci interaction for SP.

## Discussion

Drought tolerance is one of the major constraints towards achieving full yield potential from improved groundnut cultivars. In depth understanding of this complex trait and its component traits will be beneficial in order to develop stress resilient cultivars through different crop improvement approaches. Getting precise and consistent phenotyping data on such complex traits in each varied season is very difficult due to highly fluctuating environmental and soil conditions. Selections and release of new varieties based on unreliable and inconsistent phenotyping data often leads to failure in adoption by the farmers. Under such circumstances, integration of genomics tools with ongoing genetic improvement programme will be key to develop improved cultivars with high yield under drought stress conditions. In this context, effort was made through present study to identify genomic regions associated with drought tolerance related traits which can be deployed in improving pod yield under drought stress through GAB. The results indicated involvement of several M-QTLs and E-QTLs for drought tolerance related traits for Niger and Senegal.

### Phenotypic variation and broad-sense heritability

The results showed large variation within the RILs across the traits and the experiments. A significant decrease in PYLD, HYLD and HI was observed under WS condition while small or no decrease was observed for other traits. More interestingly, pod yield decreased due to water stress by 17, 41 and 37 % in the experiments conducted at Bambey during 2009 and Sadore during 2009 and 2010, respectively. Above results indicate that there was less water stress at Bambey compared to experiments conducted at Sadore. The Sadore experimental location had higher minimum temperatures and lower maximum air humidity leading to comparable lower water stress than Bambey. The above results clearly showed that variable microenvironments lead to different level of water stress which resulted in wide range of yield losses in these regions.

Varied heritability was observed for different traits across different water regimes and the experiment locations. It was interesting to note that the trait heritability for PYLD, HYLD, SCMR and HI was lower under WS than WW at Bambey location during 2009. More interestingly, the above trend was in reverse direction at Sadore during 2009 and 2010. Similar results for heritability (*H*^*2*^) in different locations were also reported for physiological, yield and yield related traits in groundnut (Painawadee et al. 2009). Not only groundnut, such results were also been reported for grain yield under WS condition in two important cereal crops i.e., rice (Lanceras et al. 2004) and wheat (Eid 2009). The heritability either remained unchanged or increased in case of SP, 100 KW and SMK % under WS condition at Bambey during 2009. This result indicated that traits such as PYLD, HYLD and HI were more prone and easily affected by water stress than SP, SMK % and 100 KW. Similarly, the heritability of the SCMR was found quite similar across different experimental sites and water regimes. Similar observations were reported in earlier experiment in groundnut (Krishnamurthy et al. 2007) which indicates that SCMR is also less prone to water stress. The above results confirm the current understanding that the heritability of complex traits show more fluctuations with the varied environmental conditions as compared to simple inherited traits. Because of this reason, the breeding lines selected in a particular location/year do not hold true in another location/season. These circumstances pose a big challenge to breeders in developing resilient crop varieties with stable performances, which ultimately limits the adoption and spread of newly developed cultivars.

### Phenotypic correlations and factors contributing to pod yield

Results showed strong significant positive correlation between two important traits i.e., PYLD and HYLD across the experiments and water regimes. In addition, PYLD and HYLD showed significant and positive correlations with the morphological traits (PH and PBr). These correlations indicate increased pod and haulm yield due to increased plant height and number of primary branches. In all the three experiments, HYLD showed strong negative correlation with HI and SMK % in both the water regimes. In this study, no correlation between SCMR and PYLD was found in any of the experiment and water regimes. It indicates no direct role of SCMR towards PYLD. As expected, the correlation between HI and PYLD was found to be higher under WS condition than under WW condition. This may be due to better allocation of carbohydrates for pod filling in groundnut under WS condition. This correlation was reinforced by the positive and significant correlation between the HI and the SP (i.e. the ratio of the kernel weight to pod weight). Lanceras et al. (2004) observed in rice that the correlation between grain yield and HI increased with the increase in water stress levels. No significant change was observed for remaining traits.

Consistently, there was a significant and positive correlation between PYLD under WW and PYLD under WS. Therefore, good agronomic performance under water stress condition could be partially explained by good potential yield. This was in agreement with previous studies in groundnut (Songsri et al. 2008a, b; Painawadee et al. 2009; Ntare and Williams 1998; Hamidou et al. 2012). Furthermore, this study showed highly significant and positive relationship for all the six traits (PYLD, HYLD, HI, SMK %, SP and SCMR) across water regimes and locations. Consistently over all the experiments, correlation was always lower for PYLD as compared to other traits indicating the highly influential nature of this trait.

The pod yield under water-limited condition was mainly determined by the HI and in some extent by the above biomass (HYLD). We observed a significant and positive relationship of HI with SMK % and SP. This indicates that the genotypes with better efficiency of translocation would have a better pod maturity percentage and/or a higher shelling percentage. In this study, we also noted a very highly significant (P < 0.001) and negative correlation between the HI under WS condition and the PBr under WW condition. From a breeder’s point of view, HI can serve as a means to predict yield and seed quality in groundnut. This result is in agreement with previous report on groundnut (Ratnakumar and Vadez 2011; Hamidou et al. 2012). These results are consistent with those reported in other crop species such as maize (Edmeades et al. 1999; Lafitte and Edmaedes 1997) and rice (Lanceras et al. 2004). Contrary to the earlier study (Painawadee et al. 2009) that reported correlation between SCMR and PYLD under WS condition, we did not found any significant correlation. However, we found a significant and positive correlation between HI and SCMR which indicates HI may have an indirect correlation with PYLD under WS via its effect on the HI.

The performance of RILs varied significantly between different locations (Fig. 2) as well as between seasons of the same location (Fig. 3). This is due to large genotype by environment (G × E) interactions for complex traits such as PYLD and HYLD. Therefore, since a combined environment QTL analysis was not logical under such circumstances, QTL analysis was carried out for each single environment. These results also caution selecting drought tolerant genotypes across locations with such large variations (Wright et al. 1996; Hamidou et al. 2012).

### Identification of location specific QTLs

#### Main-effect QTLs (M-QTLs)

QTL analysis resulted in identification of a total of 51 main-effect QTLs (M-QTLs) for nine different traits under WS condition (27 M-QTLs) and WW condition (24 M-QTLs). Many QTLs detected for different traits were overlapping in some specific genomic regions. The PVE of these M-QTLs were low indicating minor effect QTLs. Small-effect QTLs for complex traits such as yield were also been identified in rice (*Oryza sativa* L.) (Zhou et al. 2011) and maize (*Zea mays* L.) (Ribaut et al. 1997). These results confirm the current understanding that the complex traits are highly polygenic in nature and are the outcome of several small-effect genetic factors. Indeed the contribution of each locus may be negligible but the total contribution is usually significant.

Of the total QTLs identified, a QTL for SCMR detected at Bambey on the LG_AhVIII coincided with a QTL detected at Patancheru (India) by Ravi et al. (2011) using the same mapping population. Another QTL for SCMR detected at both Sadore 2009 and 2010 was also detected at Patancheru (India) by Ravi et al. (2011). No consistency for QTLs across locations and years was observed for any trait considered in this study. Such inconsistency of the QTLs between water regimes, locations and years was also previously reported (Ribaut et al. 1997; Martin et al. 1994). The results indicated inconsistency of QTLs of a given trait between experiments even under quite similar conditions. This inconsistency across seasons and locations was due to presence of high environmental influence in addition to varied soil moisture conditions. It was observed that the G × E variance component was larger than genotypic variance under water stress condition and been observed previously in groundnut (Hamidou et al. 2012), rice (Pantuwan et al. 2002) and tropical maize (Cooper et al. 2006). In addition to climatic conditions, which were quite different from one experiment to another in this study, the physical and chemical soil properties may have a great effect on the G × E of the yield and its components. These results suggest there is a need to understand the cause for these interactions.

#### Pleiotropism and linked genes

Most importantly, this study identified genomic regions containing several QTLs for different yield and yield component traits i.e., QTL clusters at several specified genomic regions. Despite significant correlations between different traits, common QTL genomic regions were not identified for highly correlated traits indicating distribution of these small-effect QTLs across the genome. If pleiotropism is the major reason, the coincidence of both the locations of QTLs for related traits and the directions of their effects can be expected. In the study, therefore, pleiotropism would explain the relation between PH and the HYLD under WW condition as indicated in LG_AhVI, LG10 and LG_AhXVII. If the simple close linkage is the major reason, the directions of the genetic effects for different QTLs may be different, although the coincidence of the locations can still be expected (Zhuang et al. 1997). It seems to be the case in our study between the QTL for the SMK % and PBr under WW condition on the LG_AhIX, between the QTL for SP under WS and the QTL for PH under WW. The detection of putative QTLs for different traits, in common regions, under the same or different environmental conditions, highlights the relation between these parameters and suggests that these regions are involved in several aspects of the determinisms to drought tolerance. Classical quantitative genetics assumes that trait correlation can be attributed to the effect of pleiotropy or to the tight linkage of genes (Hittalmani et al. 2003).

#### Epistatic effect QTLs (E-QTLs)

The epitasis analysis with QTLNetwork revealed additive × additive interactions for different traits while the GMM software revealed two-loci and three-loci interactions for some traits. It was important to note that the same two-loci interaction for SCMR (between a locus on LG_AhI and a locus on LG_AhXIII) was detected by both the softwares (QTLNetwork and GMM). Similar to M-QTLs, the E-QTLs also showed low PVE range indicating minor E-QTLs and also been earlier detected at Indian conditions (Ravi et al. 2011). The low PVE is adding weight to evidence that all these traits are quantitatively inherited including the morphological traits. In addition, inconsistency of the E-QTLs between locations and years was also observed similar to M-QTLs. Isleib et al. (1978) previously reported inconsistency of allelic interactions detected between locations and years. This result is also in agreement with the findings of Upadhyaya and Nigam (1998) who reported epistasis in 11 vegetative and reproductive traits in groundnut and interactions between epistasis and environment using a triple test cross.

## Conclusions

Heritability and correlation information provide guidelines for selection of traits in crop improvement. Among the traits analyzed under water stress condition, the harvest index and the haulm yield were more correlated with the pod yield and have intermediate broad sense heritability. Based on QTL analysis, all the nine traits analyzed were controlled by additive and multiplicative effects. The QTLs detected explained a low proportion of the phenotypic variance. Some of these QTLs over-lapped with previously reported drought tolerance QTLs detected in this population. Therefore, ignoring E-QTLs could result in significant bias which may limit progress in any breeding program for these traits. Fortunately, new approach like marker-assisted recurrent selection (MARS) and genomic selection (GS) allowed mining and advancing best lines to avoid as much as possible the effects of undesirable alleles.

## Electronic supplementary material

Supplementary material 1 (XLSX 13 kb)
